# Immunotherapy enhances the risk of tumor oxidative stress and metastasis in lung cancer with radiation pneumonitis

**DOI:** 10.3389/fimmu.2025.1629170

**Published:** 2025-07-24

**Authors:** Ruidi Jiao, Wenbo Xu, Kunpeng Xu, Mingxia Zhang, Weilong Liu, Wei Jiang, Luhua Wang

**Affiliations:** ^1^ School of Medicine, Southern University of Science and Technology, Shenzhen, Guangdong, China; ^2^ Department of Radiation Oncology, National Cancer Center/National Clinical Research Center for Cancer/Cancer Hospital & Shenzhen Hospital, Chinese Academy of Medical Sciences and Peking Union Medical College, Shenzhen, Guangdong, China; ^3^ The Second Hospital & Clinical Medical School, Lanzhou University, Lanzhou, Gansu, China; ^4^ Shenzhen Key Laboratory of Infection and Immunity, Shenzhen Third People’s Hospital, Shenzhen, Guangdong, China

**Keywords:** radiation pneumonitis, immunotherapy, metastasis, oxidative stress, lung cancer

## Abstract

Radiation pneumonitis (RP) is a prevalent complication associated with lung cancer radiotherapy; nonetheless, its effects on lung cancer immunotherapy and the underlying biological mechanisms remain inadequately elucidated. Utilizing mouse models of RP and orthotopically lung cancer, we witnessed immunotherapy-enhanced liver metastasis of lung cancer within the context of RP, accompanied by increased neutrophil infiltration of the primary tumor. Analysis of metabolic adaptations driven by the inflammatory microenvironment during treatment revealed that RP and immunotherapy act synergistically to exacerbate lipid metabolic dysregulation and oxidative stress. Integrating clinical validation with single-cell RNA sequencing data from a multicenter lung adenocarcinoma cohort, we demonstrated that elevated oxidative stress scores within tumor tissue were significantly associated with both diminished response to immunotherapy and unfavorable clinical outcomes. These findings coincided with alterations in the tumor immune microenvironment, notably a marked increase in neutrophils and activated mast cells. This investigation highlights that RP is not merely a toxicity but an active modulator of the tumor-immune-metabolism landscape. By dissecting the RP-ICB-metabolism axis, we have elucidated a novel mechanism underlying immunotherapy resistance, offering new insights into the rational design of optimized radioimmunotherapy.

## 1 Introduction

Lung cancer persists as the most prevalent malignancy and leading cause of cancer-related mortality worldwide (1, 2). Radiotherapy and immune checkpoint inhibitors (ICB) have emerged as pivotal therapies for lung cancer, demonstrating synergistic effects when combined across early to advanced disease stages (3, 4). However, this combination is tempered by exacerbated toxicities, particularly pneumonitis—a life-threatening inflammatory complication (5–7). Our prior work revealed that ICB amplifies radiation pneumonitis (RP) severity through the γδT/IL-17/neutrophil axis, while both our clinical data and prior studies consistently link treatment-related pneumonitis to diminished progression-free survival in lung cancer patients receiving combinatorial radioimmunotherapy (8–10). Preclinical study indicates no impact of RP or ICI-related pneumonitis on tumor metastasis, but radioimmunotherapy-related pneumonitis promotes it (our unpublish data). These findings underscore the urgent need to elucidate how treatment related pneumonitis dynamically reshape tumor microenvironment (TME) to drive therapeutic resistance and metastatic progression, thus identify potential therapeutic targets to mitigate these effects.

Emerging evidence implicates metabolic reprogramming as a hallmark of RP pathogenesis (11, 12). Wiebe et al. identified radiation-induced perturbations in serine and histidine metabolism within inflamed lungs, metabolites that may orchestrate immunoinflammatory cascades (13). Furthermore, metabolic signatures in lung adenocarcinoma correlate significantly with ICB responsiveness and survival outcomes (14, 15). These findings collectively posit that pneumonitis-associated metabolic dysregulation may propagate into tumor niches, yet whether and how such metabolic crosstalk impacts treatment efficacy remains unexplored. Resolving this knowledge gap demands systematic interrogation of spatiotemporal metabolic rewiring at the treatment related pneumonitis -tumor interface.

This study integrated orthotopic lung cancer mouse models with RP induction to dissect microenvironment-driven metabolic adaptations during ICB. We demonstrated that immunotherapy, in the context of RP, may synergistically polarize tumors toward a lipid-peroxidation-enriched state, exacerbate oxidative stress, and fuel liver metastasis. We retrieved a set of oxidative stress-related genes (OSRGs) from the Genecards database (https://www.Genecards.org). Clinical validation across multicentric cohorts and single-cell RNA sequencing (scRNA-seq) analyses revealed that elevated OSRGs scores inversely correlated with immunotherapy response and prognosis in lung adenocarcinoma (LUAD) patients. By unraveling the RP-ICB-metabolism axis, this work not only deciphers a previously unrecognized mechanism of immunotherapy resistance but also provides actionable targets to refine combinatorial radioimmunotherapy strategies.

## 2 Materials and methods

### 2.1 Cell culture

Lewis lung carcinoma (LLC) cells (gift from C. Li, Chinese University of Hong Kong) were confirmed mycoplasma-negative (Beyotime, China) and cultured in Dulbecco’s Modified Eagle Medium (Gibco, USA) supplemented with 10% fetal bovine serum (Gibco, Brazil) and 1% penicillin/streptomycin (Fdbio science, China) at 37°C with 5% CO2.

### 2.2 Experimental animals

Female C57BL/6N mice (5–6 weeks old, Zhejiang Weitong Lihua) were housed in a specific pathogen-free setting (23 ± 1°C, 55% humidity, 12-hour light/dark cycle) with ad libitum access to irradiated chow and autoclaved water. All procedures complied with ARRIVE guidelines and were approved by the Animal Ethics Committee of Shenzhen Third People’s Hospital (Protocol #2024-001).

### 2.3 RP model

Radiation delivery was performed using an X-RAD SmART Irradiator (Precision X-ray, North-Branford, CT, USA), equipped with cone-beam computed tomography (CBCT) capabilities, delivering 225-kV photon beams at a dose rate of 4.62 Gy/min. Mice were randomly assigned to receive either sham irradiation (0 Gy) or 24 Gy in three daily fractions (8 Gy/fraction) to the left lung.

### 2.4 Orthotopic lung cancer model establishment

Six days post-radiation, 3.75×10^4^ LLC cells in 25 μL PBS/Matrigel (ABW, Shanghai, China; 1:1 v/v) were stereotactically injected into the left lung parenchyma. Tumor growth was confirmed by computed tomography (CT) scan at day 6.

### 2.5 Immunotherapy and monitoring

Anti-mouse PD-1 (Bio X Cell, West Lebanon, NH, USA) was administered intraperitoneally (200μg/mouse, i.p., twice weekly) starting at day 7 post-implantation. Tumor volume (V) was calculated using the formula V = π/6 × L × W × H from CT scan at day 14 and 21, based on the tumor’s maximum length (L), width (W), and height (H) (16).

### 2.6 Histopathology, immunohistochemistry and immunofluorescence

RP was assessed at 7 days post-tumor implantation (pre-immunotherapy). Left lungs were fixed in 10% neutral-buffered formalin (Solarbio, China) for 24 hours, paraffin-embedded, sectioned at 4μm thickness, and stained with hematoxylin & eosin (H&E). RP severity was graded by two board-certified pathologists (blinded to treatment groups) based on the percentage of lung parenchyma affected: 1 score, less than 25%; 2 score, 25-50%; 3 score, more than 50% (8).

Neutrophil quantification was performed post-treatment completion through standardized Ly6G immunohistochemistry. Tissue sections underwent EDTA-based antigen retrieval (pH 9.0, 98°C), sequential blocking (3% H_2_O_2_-methanol/5% BSA), overnight anti-Ly6G antibody incubation (Servicebio GB11229, 1:400, 4°C), and PV-9000/DAB detection (Zhongshan Goldenbridge, China) with hematoxylin counterstain. Five random fields of view were photographed at 40x, and quantitatively analyzed for Ly6G-positive area percentage using ImageJ 1.54m software.

Arginase 1 (Arg1) is a well-established marker of the pro-tumor phenotype of neutrophils (17). To quantify Arg1^+^ Ly6G^+^ cells in tumor tissue following immunotherapy, we performed tyramide signal amplification (TSA) immunofluorescence staining. Briefly, after antigen retrieval and blocking, tissue sections were incubated overnight with the anti-Arg1 antibody (Servicebio GB115724, 1:2500, 4°C). The following day, secondary antibody (Servicebio GB23303, 1:500) and TSA reagent (Servicebio G1223, 1:500) were sequentially added. Subsequently, after repeat antigen retrieval and blocking, the sections were incubated overnight with the anti-Ly6G antibody (Servicebio GB11229, 1:500, 4°C). On the third day, secondary antibody (Servicebio GB22403, 1:200) and DAPI (Servicebio G1012) were sequentially applied. The number of Arg1^+^Ly6G^+^cells was then quantified in three fields under 20X magnification using an LSM900 confocal laser scanning microscope (ZEISS, Germany).

In addition, liver samples were prepared for paraffin embedding. Serial sections were examined, and the section exhibiting the maximal cross-sectional area of the liver was selected for H&E staining. Liver metastases in this section were then quantified.

### 2.7 Untargeted metabolomics profiling

Tumors (n = 6–7/group) were snap-frozen in liquid nitrogen, homogenized in 80% ice-cold methanol (−20°C) and centrifuged (14,000 rpm, 15 min, 4°C). Lyophilized supernatants were reconstituted in 50% ice-cold methanol and analyzed using an ACQUITY UPLC System I Class (Waters, USA) coupled to a Q-Exactive Plus high-resolution mass spectrometer (Thermo Fisher Scientific, Germany). Chromatographic separation was performed on a T3 column (100 × 2.1 mm, 1.8 µm) at 40°C with a mobile phase of (A) 5 mM ammonium acetate/5 mM acetic acid in water and (B) acetonitrile, delivered at 0.3 mL/min. The gradient program was as follows: 5% B (0-0.3 min), 5-70% B (0.3–2 min), 70-99% B (2-6.2 min), 99% B (6.2-7.5 min), 99-5% B (7.5-8.0 min), 5% B (8.0-10.0 min).

Mass spectrometry data were recorded in both positive (+4000 V) and negative (-2800 V) ion modes, converted to mzXML format and analyzed for peak extraction, retention time adjustment, metabolite annotation, and quantification. Gene Set Enrichment Analysis (GSEA) was performed using the Kyoto Encyclopedia of Genes and Genomes (KEGG) database to assess intergroup metabolic pathway differences. To identify significant metabolic alterations, we employed a combination of univariate and multivariate statistical analyses. Initially, t-tests were performed to evaluate the statistical significance of intergroup differences in metabolite abundance. Subsequently, partial least squares discriminant analysis (PLS-DA) was conducted, and variable importance in projection (VIP) scores were extracted for each metabolite. Significantly altered metabolites were defined as those with fold change (FC) > 1.20 or < 0.83, VIP > 1, and *P*-value < 0.05. Significantly altered metabolites were then used for the KEGG enrichment analysis.

### 2.8 ROS detection

LLC cells (1 × 10^5^/well) were treated for 24 hours with linoleic acid (low-dose: 50 μM; high-dose: 100 μM; Sigma-Aldrich, Germany). Intracellular ROS was visualized using a ROS Assay Kit (Beyotime, China) and imaged via confocal laser scanning microscopes (Carl Zeiss, Germany).

### 2.9 Transcriptomic data acquisition

Public datasets included two scRNA-seq datasets related to immunotherapy in LUAD: GSE207422 (Gene Expression Omnibus [GEO], https://www.ncbi.nlm.nih.gov/geo) and HRA002509 (Zenodo, https://zenodo.org/). Bulk RNA-seq datasets comprised TCGA-LUAD (The Cancer Genome Atlas [TCGA], https://portal.gdc.cancer.gov), GSE30219, GSE31210, GSE72094, GSE126044, GSE207422, OAK, and POPLAR, with the latter four being immunotherapy cohorts. OAK and POPLAR were obtained from previous study (18). By searching the Genecards database with the term “oxidative stress”, we identified 1663 OSRGs with a relevance score ≥ 3. Detailed gene information is available in **Supplementary Table 1**.

### 2.10 OSRGs score development

Two scRNA-seq datasets (GSE207422 and HRA002509) were integrated using Seurat v4. Low-quality cells (<200 genes/cell) and genes expressed in <3 cells were filtered. Batch effects were mitigated during integration using the SCTransform method (https://github.com/ChristophH/sctransform) with default parameters. Following integration, principal component analysis was performed on the scaled and centered data, and the top 30 principal components were used for dimensionality reduction via Uniform Manifold Approximation and Projection (UMAP). Cells were annotated based on the expression of known marker genes from the original publications.

Based on pathologic response to immunotherapy, patients were classified as responders (major pathologic response, MPR) or non-responders (non-MPR, NMPR). We identified differentially expressed genes (DEGs) significantly upregulated (logFC > 0.25, adjusted *P* value < 0.05) in each cell subset of non-responders relative to responders. By intersecting these DEGs (GSE207422 and HRA002509) and the OSRGs gene set, we identified OSRGs potentially linked to immunotherapy response. The optimal modeling algorithm was explored and validated across six bulk RNA-seq cohorts (TCGA-LUAD, GSE30219, GSE31210, GSE72094, OAK, POPLAR) using 118 machine learning algorithms. Random survival forest (RSF) analysis of TCGA-LUAD data (R software, randomForestSRC package) was then used to assess the relationship between the selected genes and prognosis.

### 2.11 OSRGs score-immune correlation

The AUCell algorithm quantified OSRGs activity in cell subpopulations and tumor tissue spatial transcriptomic sections. Correlations between OSRGs score and immunotherapy response were validated in four bulk RNA-seq cohorts (GSE126044, GSE207422, OAK, POPLAR), and neutrophil infiltration in the TME was assessed via CIBERSORT in TCGA-LUAD cohort. LUAD patients were stratified into high/low OSRGs score groups (median cutoff) across four cohorts (TCGA, GSE31210, OAK, and POPLAK). Kaplan-Meier analysis evaluated survival differences. Immune cell fractions (22 subsets) were deconvoluted using CIBERSORT in TCGA-LUAD databases, and pathway activity was profiled via the R package ‘GSVA’ (v 1.30.0).

### 2.12 Statistical analysis

Statistical analyses used Mann-Whitney U tests for group comparisons (*P* < 0.05), with data presented as mean ± SEM (standard error of the mean). Analyses were performed in GraphPad Prism 8 (San Diego, California, USA) and R v4.3.1.

## 3 Results

### 3.1 Immunotherapy promotes liver metastasis of lung cancer under RP

**Figure 1:**
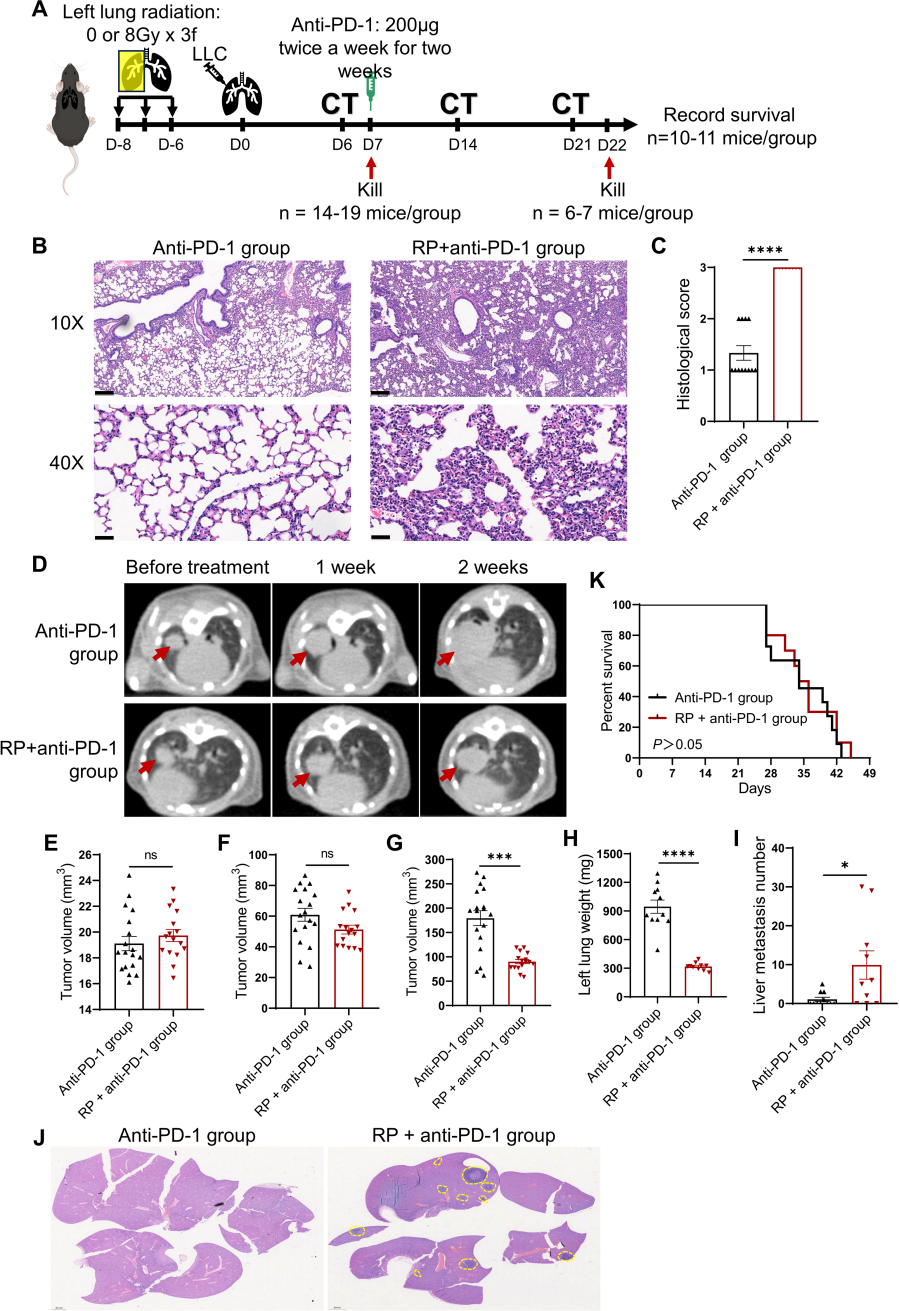
Immunotherapy promotes liver metastasis of lung cancer under radiation pneumonitis. **(A)** Experiment flowchart. C57BL/6N mice received fractionated left lung radiotherapy (anti-PD-1 group: sham irradiation [0Gy×3 fractions]; RP+anti-PD-1 group: 8Gy×3 fractions). Six days post-radiotherapy, 3.75 × 10^4^ LLC cells were orthotopically injected into the left lung. Tumor establishment was confirmed by CT imaging at day 6 post-implantation. Then mice were sacrificed for pneumonitis assessment (n = 8-12/group). Anti-PD-1 therapy was injected intraperitoneally twice weekly for two weeks at day 7. CT imaging was performed once a week during the treatment. **(B)** Representative H&E-stained lung sections pre-immunotherapy. Scale bar, 200 µm (upper), 50 µm (lower). **(C)** Semiquantitative pneumonitis scoring. **(D)** Longitudinal CT imaging (red arrows: primary tumors). **(E-G)** Tumor volumes dynamics pre-treatment, 1 week, and 2 weeks post- immunotherapy. **(H-J)** Tumor-bearing lung weight, the number of liver metastases, and representative H&E-stained liver sections (yellow dashed lines: metastatic foci; scale bar: 2 mm). **(K)** Kaplan-Meier survival curves. Data represent mean ± SEM. ns, not significant, *P<0.05, ***P<0.001, ****P<0.0001 (Mann-Whitney U Test).

The animal experimental workflow was illustrated in **Figure 1A**. Mice receiving fractionated left lung radiation exhibited marked histopathological features of RP at 7 days post-tumor implantation, including significant diffuse congestion, alveolar wall thickening, and inflammatory cell infiltration (**Figure 1B**). Tissue injury scores were significantly elevated in irradiated lungs compared to unirradiated lungs (**Figure 1C**). These findings established an experimental basis for our subsequent investigation of the impact of RP on lung cancer immunotherapy.

Longitudinal CT imaging revealed no significant difference in primary tumor volume between the two groups at baseline or week 1 post-treatment (**Figures 1D-F**). By week 2, mice in the RP + anti-PD-1 group showed suppressed primary tumor growth (*P* < 0.05, **Figure 1G**), and endpoint tumor-bearing lung weight was reduced in the RP + anti-PD-1 group (**Figure 1H**). However, mice in the RP + anti-PD-1 group developed significantly higher incidence (70.00% vs. 36.36%, *P* > 0.05) and burden (1.09 ± 1.76 vs. 9.90 ± 11.52, *P* < 0.05) of liver metastases (**Figures 1I, J**). Consequently, survival outcomes were comparable between the anti-PD-1 group and the RP + anti-PD-1 group (median survival: 34 vs. 35 days, *P* > 0.05, **Figure 1K**).

### 3.2 Immunotherapy induces unique tumor metabolic reprogramming in the context of RP

**Figure 2:**
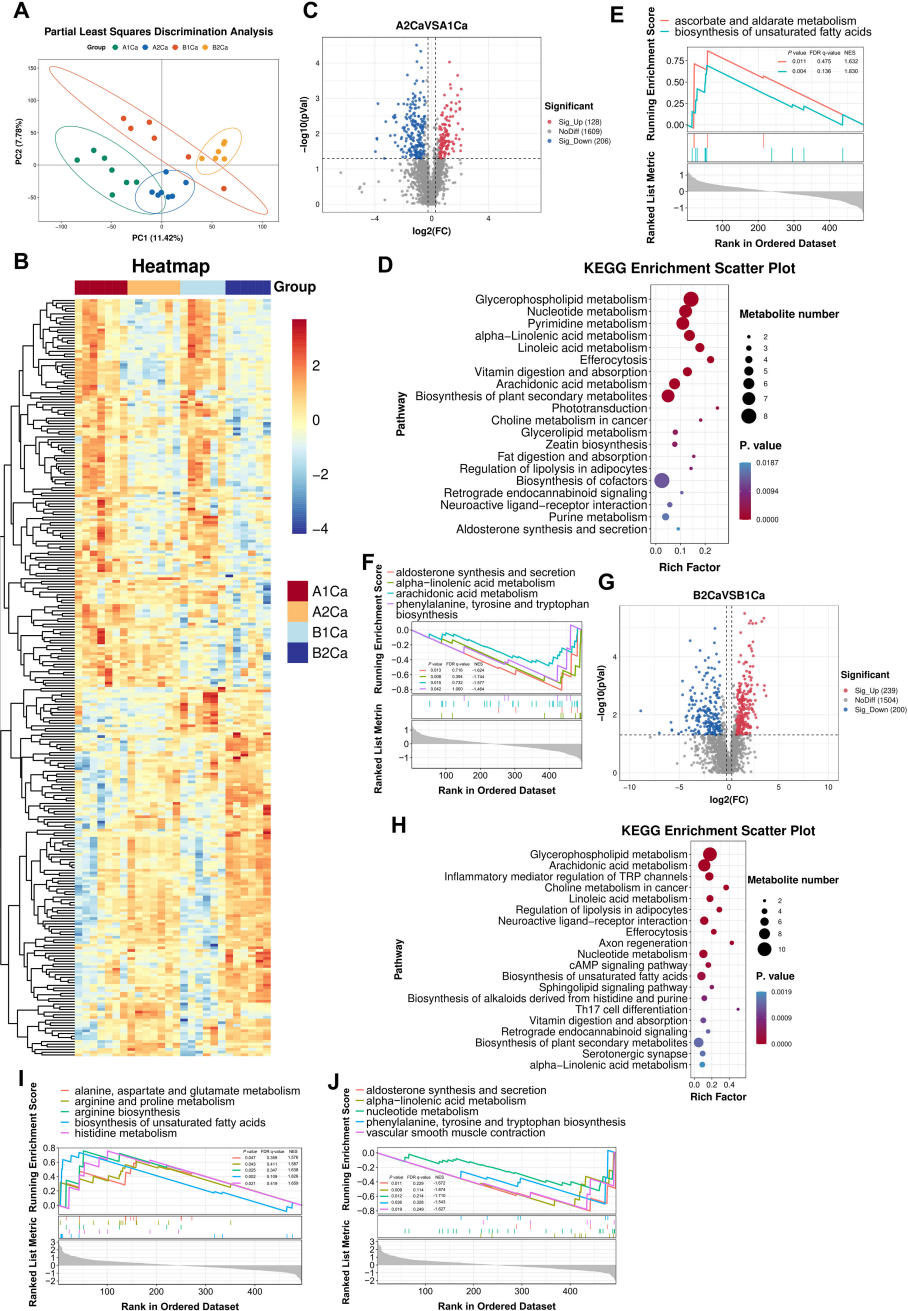
Radiation pneumonitis potentiates immunotherapy-induced metabolic rewiring in lung tumors. **(A)** Partial Least Squares Discrimination Analysis of tumor metabolites shows distinct clustering between anti-PD-1group (A1Ca: pre-treatment; A2Ca: post-treatment) and RP + anti-PD-1 group (B1Ca: pre-treatment; B2Ca: post-treatment). **(B)** Representative metabolic profiles of the two groups. **(C, D)** Volcano plots and KEGG enrichment analysis (top 20 pathways) of significantly altered metabolites in the anti-PD-1 group (post- vs. pre-immunotherapy). **(E, F)** Up- and down-regulated pathways by Gene Set Enrichment Analysis (GSEA) using the KEGG database in the anti-PD-1 group (post- vs. pre-immunotherapy). **(G, H)** Volcano plots and KEGG enrichment analyses (top 20 pathways) of differential metabolites in the RP + anti-PD-1 group (post- vs. pre-immunotherapy). **(I, J)** Up- and down-regulated pathways by GSEA in the RP + anti-PD-1 group (post- vs. pre-immunotherapy). FDR, false discovery rate; NSE, normalized enrichment score.

PLS-DA revealed distinct separation between two groups before and after immunotherapy (**Figure 2A**). Untargeted metabolomics identified 1943 metabolites across tumor tissue, predominantly lipids and lipid-like molecules, organic acids and derivatives, and organoheterocyclic compounds. The partial metabolic profiles of tumor tissues were shown in **Figure 2B**. Immunotherapy induced significant alterations in the metabolic landscape of tumor tissues independent of RP.

Immunotherapy alone upregulated 128 metabolites (e.g., sorbitol, ergothioneine, lysophosphatidylcholine [LPC P-18:0], uridine5-diphosphate, and sclareol) and downregulated 206 metabolites (e.g., naringenin, guanosine-5’-monophosphate, lysophosphatidylglycerol [LPG 18:0, LPG 18:1], and L-Tryptophan) (**Figure 2C**). Top 5 enrichment pathways of differential metabolites included glycerophospholipid metabolism, nucleotide metabolism, pyrimidine metabolism, alpha-linolenic acid metabolism, and linoleic acid metabolism (**Figure 2D**). GSEA further revealed activation of ascorbate/aldarate metabolism and unsaturated fatty acids biosynthesis in tumor tissues after immunotherapy (**Figure 2E**), alongside suppression of alpha-linolenic acid metabolism, aldosterone synthesis and secretion, arachidonic acid metabolism, and phenylalanine, tyrosine and tryptophan biosynthesis (**Figure 2F**).

In RP mice (**Figure 2G**), metabolic changes induced by immunotherapy were more abundant, with 239 upregulated metabolites (e.g., L-kynurenine, prostaglandin E2, histamine, lysophosphatidic acid [LPA O-18:0], cis-8,11,14,17-Eicosatetraenoic acid), and 200 downregulated metabolites (e.g., guanosine-5’-monophosphate, glutamine, 7-Oxocholesterol, LPG 16:2, LPG 17:0). Pathway analysis revealed unique enrichment in glycerophospholipid metabolism, arachidonic acid metabolism, inflammatory mediator regulation of TRP channels, choline metabolism in cancer, and linoleic acid metabolism (**Figure 2H**). GSEA demonstrated activation of biosynthesis of unsaturated fatty acids, histidine metabolism, arginine biosynthesis, arginine and proline metabolism, and alanine, aspartate and glutamate metabolism in tumor tissues after immunotherapy (**Figure 2I**), concurrent with suppression of alpha-linolenic acid metabolism, aldosterone synthesis and secretion, nucleotide metabolism, vascular smooth muscle contraction, and phenylalanine, tyrosine and tryptophan biosynthesis (**Figure 2J**). These results indicated that immunotherapy may trigger more pronounced and distinct metabolic reprogramming in the RP microenvironment.

### 3.3 Immunotherapy synergizes with RP to promote oxidative stress

**Figure 3:**
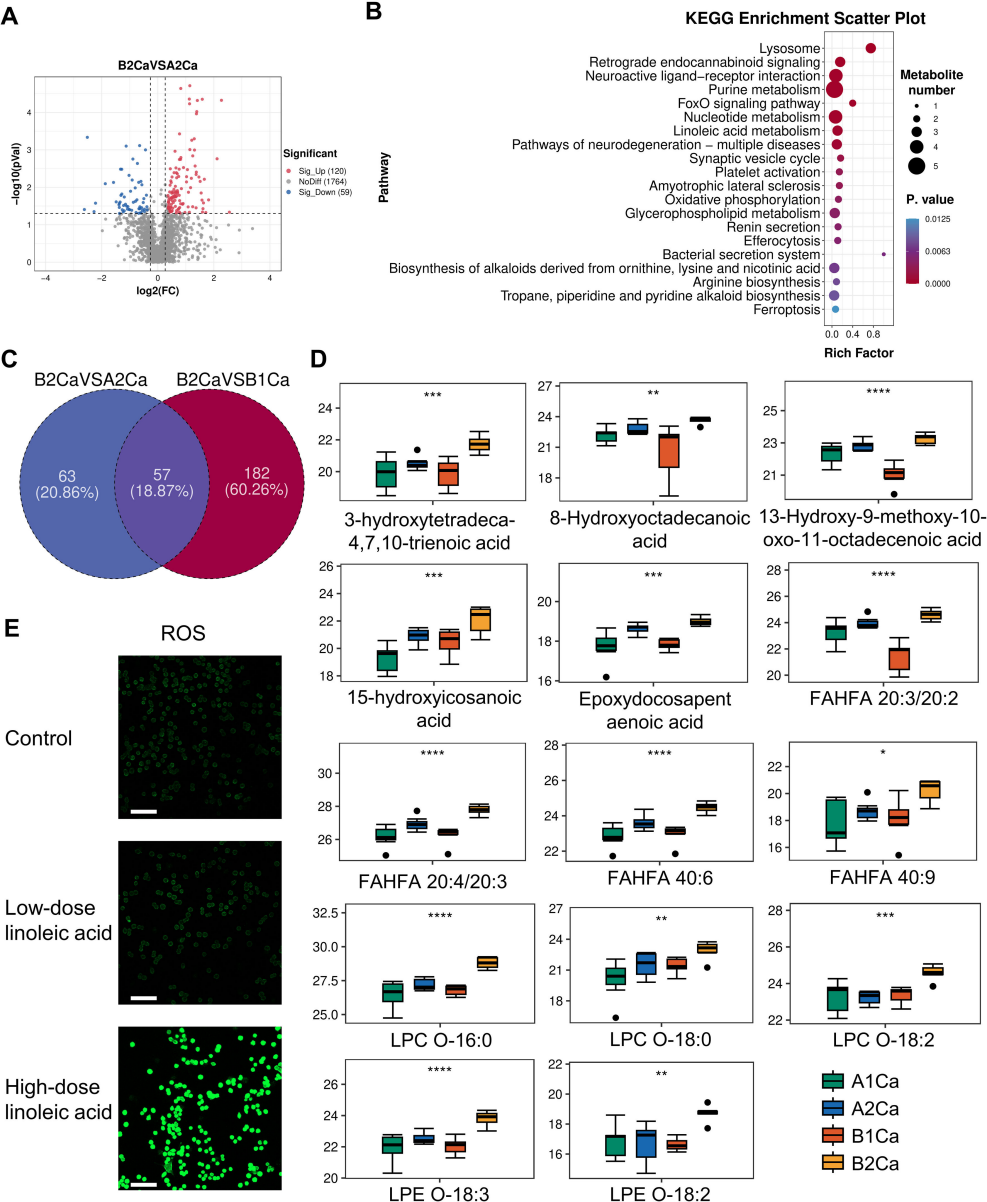
Immunotherapy synergized with radiation pneumonitis to promote oxidative stress. **(A)** Volcano plots of differential metabolites between RP + anti-PD-1 (B2Ca) and anti-PD-1 (A2Ca) groups post-immunotherapy. **(B)** Top 20 enriched KEGG pathways of the above differential metabolites. **(C)** Venn plot showing the intersection of metabolites significantly upregulated in the RP+ anti-PD-1 group post-immunotherapy compared with before treatment and metabolites significantly upregulated in **(A)**. **(D)** Relative levels of 14 oxidative stress-related metabolites pre- and post-immunotherapy in both groups. **(E)** Confocal imaging of ROS in LLC cells treated with linoleic acid. Scale bar, 100 µm. *P<0.05, **P<0.01, ***P<0.001, ****P<0.0001 by one-way ANOVA Test. FAHFA, branched fatty acid esters of hydroxy fatty acid; LPC, lysophosphatidylcholine; LPE, lysophosphatidylethanolamine.

Metabolomic profiling of tumors at 2 weeks post-immunotherapy revealed profound metabolic rewiring in RP + anti-PD-1 mice compared to anti-PD-1 group (**Figure 3A**). A total of 120 metabolites were significantly elevated, while 59 metabolites were decrease. KEGG enrichment analysis identified lysosome, retrograde endocannabinoid signaling, neuroactive ligand-receptor interaction, purine metabolism, and the FoxO signaling pathway as top altered pathways (**Figure 3B**).

Among the above 120 upregulated metabolites, 57 were also significantly elevated compared with the RP + anti-PD-1 group pre-immunotherapy, with seven metabolites—phosphatidylcholine (PC(18:1/14:0)), PC(18:0/20:5), PC(P-18:1/20:4-OH), PC(20:3/20:3), PC(18:3/18:2), PC(20:3/16:0), and cis-8,11,14-Eicosatrienoic acid—enriched in linoleic acid metabolism (**Figure 3C**). Furthermore, oxidation stress-related lipids, including hydroxy fatty acids, LPCs, branched fatty acid esters of hydroxy fatty acid (FAHFA), and epoxydocosapentaenoic acid were predominant (**Figure 3D**). This evidence indicates that the synergistic effect of immunotherapy and RP lead to severe dysregulation of lipid metabolism and pronounced oxidative stress response in the tumor tissue.

### 3.4 Linoleic acid upregulates ROS in LLC cells

Given the enrichment of linoleic acid metabolism in RP + anti-PD-1 group post-immunotherapy, we interrogated its functional role in driving oxidative stress. As expected, LLC cells treated with high-dose linoleic acid had significantly higher intracellular ROS levels compared to both control and low-dose linoleic acid (**Figure 3E**), supporting linoleic acid metabolism as a latent driver of redox imbalance in lung cancer cells.

### 3.5 Determination of the OSRGs score system

**Figure 4:**
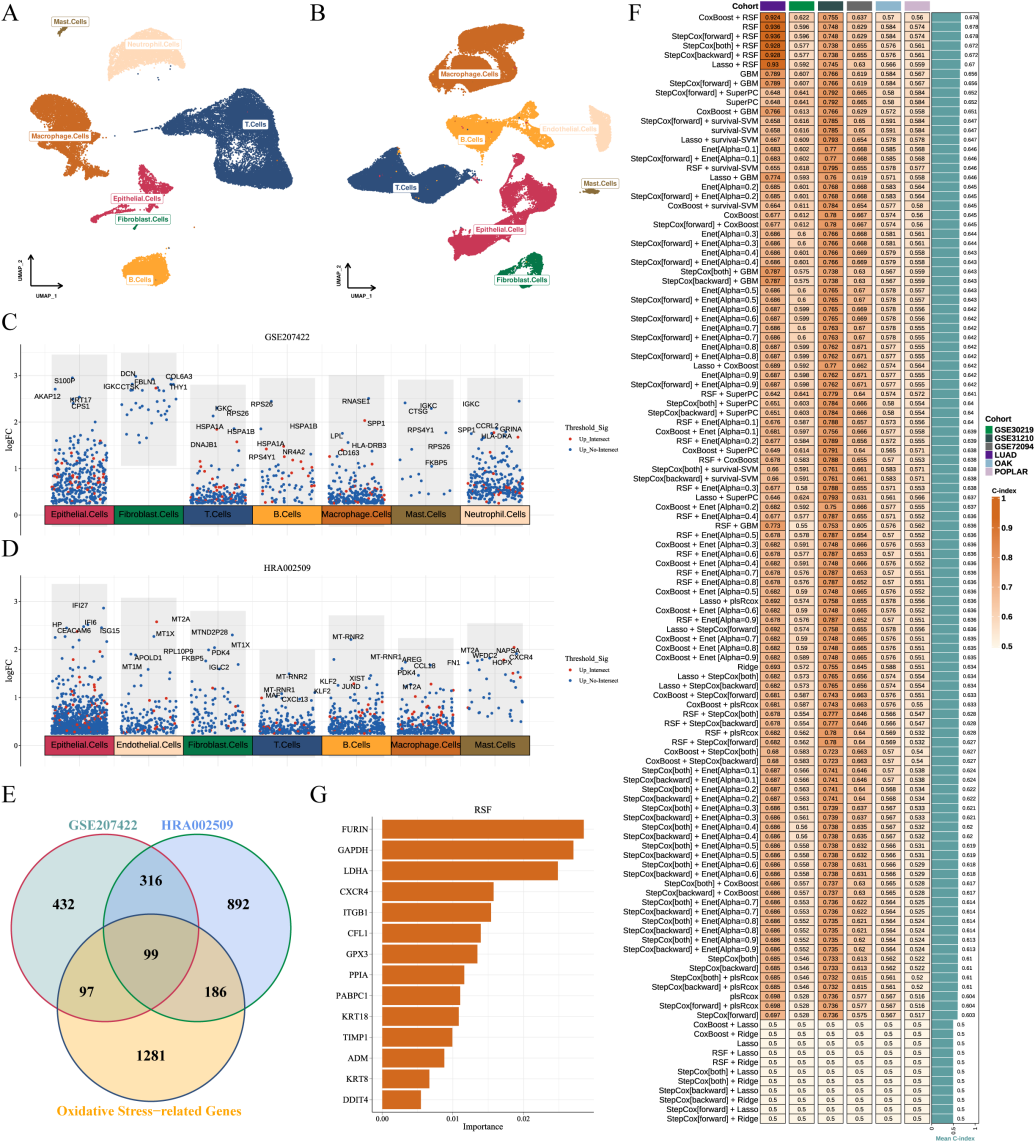
Development and validation of the OSRGs score system. **(A, B)** UMAP plots of two immunotherapy-related LUAD scRNA-seq datasets (GSE207422 and HRA002509), depicting eight major cell subpopulations. **(C, D)** Manhattan plot of significantly upregulated genes in non-responders across cell subset. Red dots indicate genes shared by ≥ 2 cell subsets; top-ranked genes per subset are annotated. **(E)** Venn diagram of differentially expressed genes from GSE207422, HRA002509, and OSRGs set. **(F)** C-index of OSRGs score system among 118 machine learning algorithms across six cohorts. **(G)** Random survival forest analysis ranks top 14 prognostic OSRGs. LUAD, lung adenocarcinoma; OSRGs, oxidative stress-related genes; UMAP, Uniform Manifold Approximation and Projection.

We integrated two scRNA-seq cohorts of immunotherapy-treated LUAD patients (GSE207422, n=15; HRA002509, n=19), resolving eight major cell types—epithelial cells, endothelial cells, fibroblasts, T cells, B cells, macrophages, mast cells, and neutrophils (**Figures 4A, B**). Comparative analysis of NMPR versus MPR identified 944 (GSE207422, **Supplementary Table 2**) and 1493 (HRA002509, **Supplementary Table 3**) significantly regulated DEGs, respectively (**Figures 4C, D**). Intersecting these DEGs with 1663 OSRGs yielded 99 candidate genes potentially implicated in immunotherapy-driven redox regulation (**Figure 4E**). Machine learning optimization across 118 algorithms identified CoxBoost + RSF as the optimal model (mean C-index = 0.678) (**Figure 4F**; **Supplementary Figure 1**, **Supplementary Table 4**). Then final OSRGs score was derived from 14 genes: OSRGs score = 0.182 × *LDHA* + 0.129 × *ITGB1 +* 0.473 × *PPIA* + 0.034 × *KRT8 +* 0.098 × *DDIT4 +* 0.171 × *FURIN* + 0.102 × *ADM* + 0.188 × *TIMP1 +* 0.090 × *PABPC1 +* 0.084 × *CFL1* - 0.026 × *GAPDH* - 0.141 × *GPX3* - 0.026 × *KRT18* - 0.185 × *CXCR4* (**Figure 4G**; **Supplementary Figure 2**).

### 3.6 High OSRGs score predicts immunotherapy resistance and immune dysfunction

**Figure 5:**
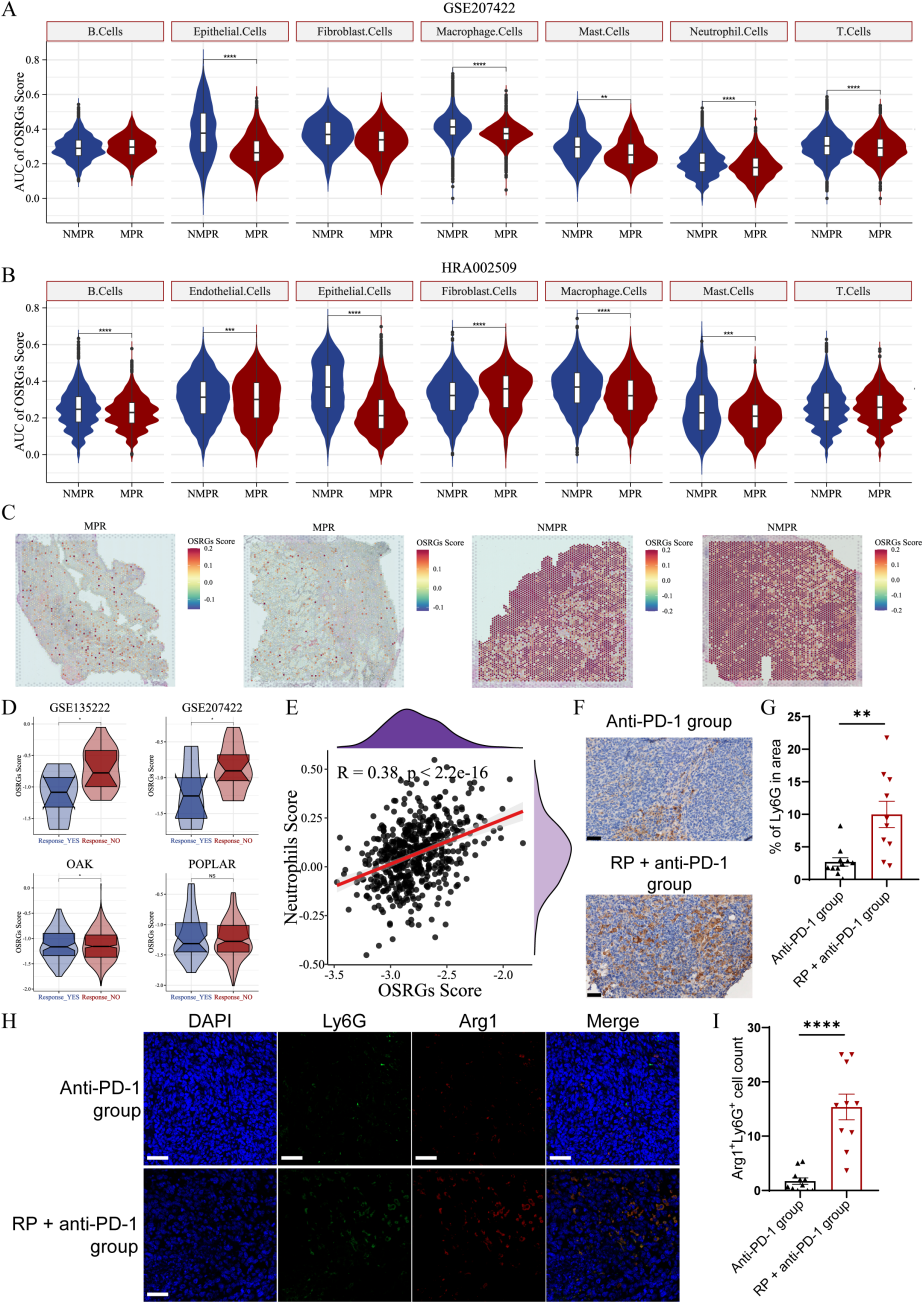
OSRGs scores correlate with immunotherapy resistance and neutrophil infiltration in LUAD. **(A)** Violin plots of OSRGs scores across cell subpopulations in the GSE207422 datasets. **(B)** Violin plots of OSRGs scores across cell subpopulations in the HRA002509 datasets. **(C)** OSRGs scores in tumor tissue sections from responder and non-responder. **(D)** Violin plots depicting OSRGs scores in tumor tissue from responder and non-responder validated across the GSE126044, GSE207422, OAK, and POPLAR datasets. **(E)** Correlation analysis between tumor tissue OSRGs score and neutrophil infiltration in the TCGA-LUAD dataset **(A–D)**: NS, not significant, *P<0.05, **P<0.01, ***P<0.001, ****P<0.0001. **(F)** Immunohistochemical staining and **(G)** quantitative analysis of tumor-infiltrated neutrophil in anti-PD-1 group and RP + anti-PD-1 group mice. Scale bar, 50 µm. **(H)** Immunofluorescence images and **(I)** quantitative analysis of Arg1 (red) and Ly6G (green) co-expressing cells in the TME of anti-PD-1 group and RP + anti-PD-1 group mice. Scale bar, 50 µm. Data represent mean ± SEM. **P<0.01, ****P<0.0001 (Mann-Whitney U Test). LUAD, lung adenocarcinoma; MPR, major pathologic response; NMPR, non-MPR; OSRGs, oxidative stress-related genes; TCGA, The Cancer Genome Atlas; UMAP, Uniform Manifold Approximation and Projection.

In scRNA-seq cohorts, the highest OSRG scores were observed in macrophages and fibroblasts (**Supplementary Figure 3**). OSRGs scores of tumor cells were significantly elevated in non-responders compared to responders (*P* < 0.0001, **Figures 5A, B**). Furthermore, this redox imbalance extended to tumor-infiltrating neutrophils, T cells, and macrophages (*P* < 0.0001, **Figures 5A, B**), suggesting broad impairment of anti-tumor immunity. Simultaneously, analysis of spatial/bulk RNA-seq among immunotherapy cohorts also revealed a relevance between high OSRGs score in tumor tissue and adverse immunotherapy response (**Figures 5C, D**). Correlation analysis revealed a positive association (R = 0.38, *P* < 0.0001) between OSRGs score and neutrophil infiltration in the TME (**Figure 5E**). Consistent with this, our *in vivo* experiments detected a marked increase in tumor-infiltrating neutrophils and the pro-tumor phenotype in the RP + anti-PD-1 group, alongside unbalanced oxidative stress (**Figures 5F-I**). These findings suggest that the interplay between oxidative stress and neutrophils may contribute to immunotherapy resistance.

### 3.7 High OSRGs score correlates with TME disorder and poor survival in LUAD

**Figure 6:**
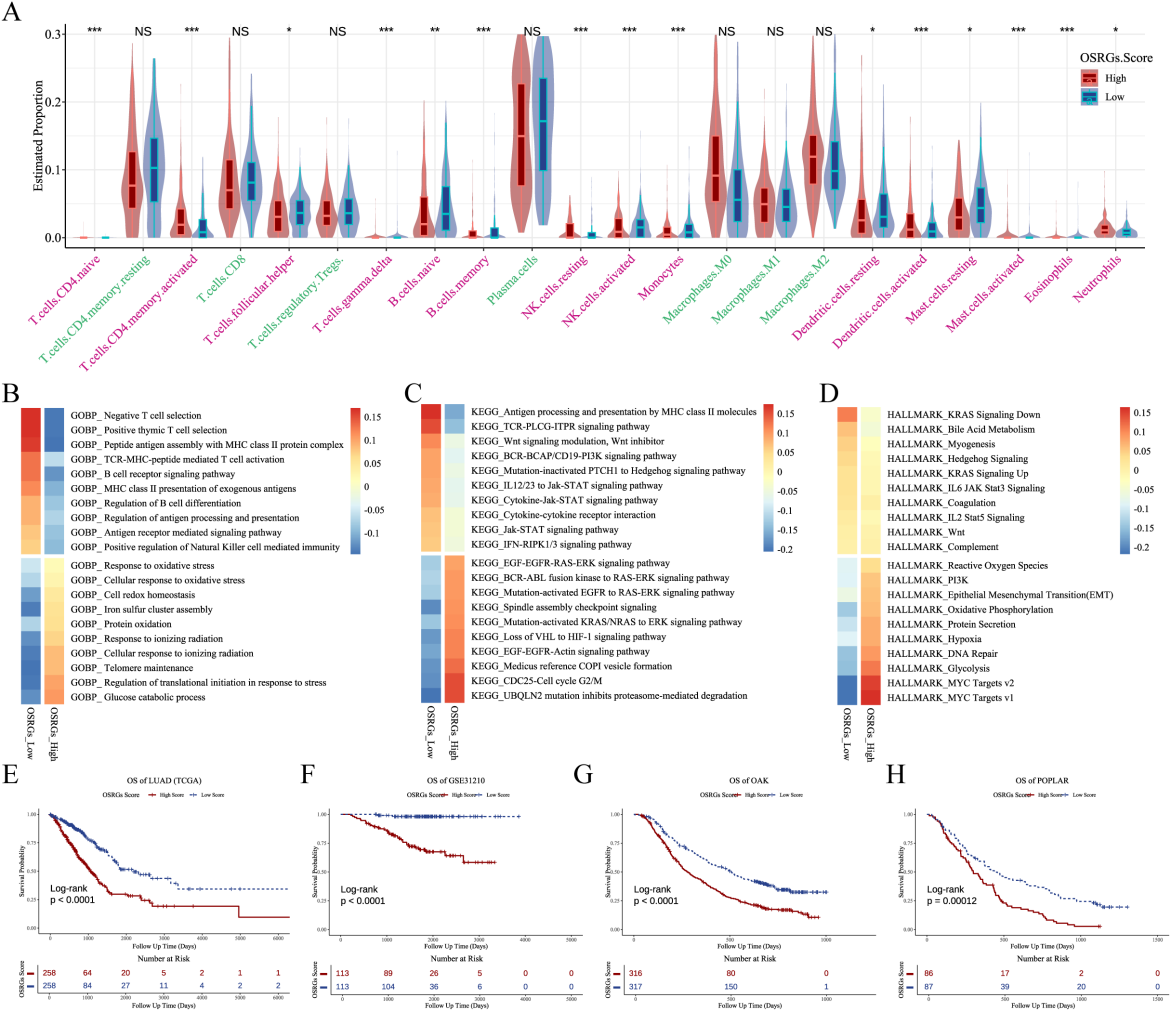
OSRGs score stratifies TME features and prognosis in LUAD. **(A)** Violin plots of immune cell deconvolution analysis of TCGA-LUAD samples stratified by median OSRGs score. **(B-D)** Pathway activity heatmap (GOBP, KEGG, and HALLMARK) across TCGA-LUAD cohort comparing high vs. low OSRGs groups. **(E-H)** Kaplan-Meier survival curves demonstrating reduced survival in high OSRGs patients across four cohorts (TCGA, GSE31210, OAK, and POPLAK datasets). LUAD, lung adenocarcinoma; GOBP, Gene Ontology Biological Process; KEGG, Kyoto Encyclopedia of Genes and Genomes; OSRGs, oxidative stress-related genes; TCGA, The Cancer Genome Atlas; TME, tumor microenvironment. NS, not significant, *P<0.05, **P<0.01, ***P<0.001.

Stratification by median OSRGs score revealed divergent TME composition between high- and low-score LUAD patients. As depicted in **Figure 6A**, high-OSRGs tumor exhibited elevated infiltration of neutrophils, activated mast cells, activated dendritic cells, activated memory CD4+ T cells, and resting NK cells, alongside reduced activated NK cells, follicular helper T cells, naive B cells, memory B cells, monocytes, and resting dendritic cells.

Functional enrichment analysis linked high-OSRGs scores to activation of response to oxidative stress, glycolysis, and EMT (**Figures 6B-D**; **Supplementary Table 5**). Oxidative stress may reshape TME into a hostile yet permissive niche that paradoxically accelerates clonal selection of evolved cancer cells. Critically, LUAD patients with high-OSRGs scores had significantly worse prognoses in the TCGA, GSE31210, OAK, and POPLAR datasets (**Figures 6E-H**).

## 4 Discussion

Metabolomic profiling has emerged as a critical tool for deconstructing therapy-induced perturbations in tumor ecosystems. By integrating preclinical models with clinical cohorts, we demonstrate that anti-PD-1 therapy synergized with RP markedly aggravates oxidative stress to establish lipid peroxidation-enriched niches in lung cancer TME, which may facilitate hepatic metastasis. High-OSRGs score associates with poor immunotherapy response and adverse survival of LUAD patient. Our findings position RP not merely as a toxicity but as an active modulator of the tumor-immune-metabolic axis, with potential implications for combinatorial radioimmunotherapy.

Dysregulated lipid metabolism, a hallmark of cancer, plays a crucial role in the process of tumor metastasis (19). Metabolic abnormalities enhance the survival capabilities of cancer cells, enabling them to adapt to adverse microenvironments (20). Furthermore, lipid metabolic reprogramming accelerates tumor progression by promoting immune evasion in cancer cells (21). Increased levels of lipid peroxidation are observed in exhausted T cells, tumor-promoting macrophages, and immunosuppressive neutrophils (22). Our study revealed elevated levels of lipid peroxidation, and linoleic acid derivatives within polyunsaturated PC, in tumor tissue within the RP microenvironment following immunotherapy. These metabolites were previously reported to be linked to progression in ovarian and hepatocellular carcinoma (23, 24). Mechanistically, cytochrome P450-mediated oxidation of linoleic acid generates bioactive epoxy fatty acid and pro-inflammatory oxylipins (e.g., 13-HODE and 9-HODE), which induce angiogenesis and foster cellular migration (25). Furthermore, consistent with the findings of our study, excess linoleic acid activated oxidative stress (26, 27). These insights illustrate the close relationship between oxidative stress and tumor metastasis. In addition, adding immunotherapy to RP also significantly boosted LPC and LPE in tumor tissues, indicating oxidative stress-mediated cell membrane damage. Zhang et al. demonstrates that LPC inhibits human lung cancer cell proliferation and induces tumor cell death, leading to reduced tumor growth and sizes in mice (28). These findings align with our observation of reduced tumor size and weight in the RP + anti-PD-1 group.

Oxidative stress is pivotal in tumor biology, driving genomic instability, inflammation, and angiogenesis, crucial for tumor growth and spread (29–31). Oxidative stress can activate protein kinase A in tumor cells, lead to the phosphorylation of RING finger protein 25, and subsequently mediate E-cadherin ubiquitination and degradation, ultimately promoting hepatocellular carcinoma metastasis (32). Furthermore, tumor stem cells exhibit a more robust antioxidant defense system compared to tumor cells (33), suggesting that a moderate oxidative stress microenvironment may facilitate the acquisition of stem-like properties in tumor cells and promote metastasis. In light of these mechanisms, we hypothesize that the oxidative stress induced by RP in synergy with immunotherapy may enhance the invasive and metastatic potential of tumor cells through the aforementioned biological processes. Our machine learning-derived OSRGs score analysis detects a close link between oxidative stress and immunotherapy resistance as well as patient survival. The association between oxidative stress and unfavorable patient outcomes was also established in gastric cancer, colorectal cancer, and glioblastoma (34–36). Strategies targeting ROS clearance and disrupting redox adaptation have shown promise in suppressing metastasis (37). For instance, fangchinoline, by promoting the degradation of NADPH oxidase 4 to reduce cytoplasmic ROS levels, reverses EMT and inhibits the invasion and migration of lung cancer cells (38). Its anti-tumor effects have been validated in animal models. The glutathione peroxidase mimic Ebselen has demonstrated inhibitory effects on colorectal cancer in both cellular and animal studies (39). Similarly, brusatol, a potent inhibitor of antioxidant response element transcription factor, can promote the ubiquitination of nuclear factor erythroid 2-related factor 2, thereby suppressing its anti-oxidative stress capacity, inhibiting metastasis, reversing drug resistance in animal tumor models, and enhancing the efficacy of chemotherapy (40). However, inhibiting antioxidant-related genes may cause adverse effects in normal cells. Therefore, careful titration of these inhibitors in combination with radiotherapy or immunotherapy is crucial to achieve optimal synergistic anti-tumor effects while minimizing toxicity to normal tissues. Future research should focus on developing more targeted redox modulators to achieve precise intervention within TME, thereby maximizing efficacy and reducing toxicity.

Tumors with high OSRGs scores exhibit a neutrophil-dominated TME characterized by lower immune cell infiltration and elevated tumor purity (41, 42). Consistently, in our study, high-OSRGs scores in LUAD tumor tissue accompany increased neutrophils and poor immunotherapy response. Concordantly, tumor-infiltrating neutrophils increased in the RP + anti-PD-1 group compared to the anti-PD-1 group. The multifaceted roles of tumor-associated neutrophils (TANs) remain a complex area requiring further exploration. TANs promote resistance through multiple mechanisms: secreting immunosuppressive cytokines to inhibit cytotoxic T lymphocyte activity, releasing neutrophil extracellular traps to shield tumors from immune surveillance, and directly escorting circulating tumor cells to promote cell cycle progression and accelerate metastasis (43–47). Recent work further implicates BHLHE40-driven glycolytic reprogramming in polarizing neutrophils toward a pro-tumor phenotype (48), highlighting metabolic targeting as a therapeutic strategy. Coincidentally, we also observed an increased pro-tumor phenotype of neutrophils within the severely dysregulated lipid metabolism TME. This also may contribute to RP-mediated immunotherapy resistance in lung cancer.

This study also has certain limitations. While our research is grounded in metabolomics, we are unable to simultaneously integrate transcriptomic data due to sample limitations. This limits a comprehensive elucidation of the molecular mechanisms through which RP influences lung cancer immunotherapy efficacy. Therefore, future research will build upon these findings and strive to mitigate the impact of treatment-related adverse events on efficacy.

In summary, this study reveals that immunotherapy, in conjunction with RP, promotes widespread and complex lipid metabolic dysregulation in tumor tissues, including elevated levels of oxidative stress-related lipids, and enhances liver metastasis in lung cancer. Furthermore, high-OSRGs scores in tumor tissues are likely associated with increased neutrophil infiltration in the TME, immunotherapy resistance, and poor patient prognosis. These findings provide a rationale for investigating the mechanisms by which RP modulates immunotherapy efficacy and for exploring oxidative stress-targeted strategies to optimize radioimmunotherapy outcomes in lung cancer.

## Data Availability

The original contributions presented in the study are included in the article/Supplementary Material. Metabolomics data have been uploaded to the OMIX repository (https://ngdc.cncb.ac.cn/omix/), data number OMIX010147. Further inquiries can be directed to the corresponding author
