# Supplementary material for: Immunotherapy enhances the risk of tumor oxidative stress and metastasis in lung cancer with radiation pneumonitis
Source: Front Immunol. 2025 Jul 24;16:1629170. doi: 10.3389/fimmu.2025.1629170 (PMC12328386; doi:10.3389/fimmu.2025.1629170)
Supplement: Supplementary file 1 [file DataSheet1.docx]

**Supplementary material**

**Supplementary Tables**

Supplementary Table 1 | List of 1663 oxidative stress-related genes (OSRGs) with a relevance score ≥ 3 obtained from the Genecards database.

Supplementary Table 2 | GSE207422: List of 944 significantly upregulated DEGs in responders.

Supplementary Table 3 | HRA002509: List of 1493 significantly upregulated DEGs in responders.

Supplementary Table 4 | The procedure for constructing the OSRGs-score system with the CoxBoost + RSF algorithm.

Supplementary Table 5 | Partial list of pathway scores (GOBP, KEGG, and HALLMARK) in high and low OSRGs-score groups within the TCGA-LUAD cohort.

**Supplementary Figures**


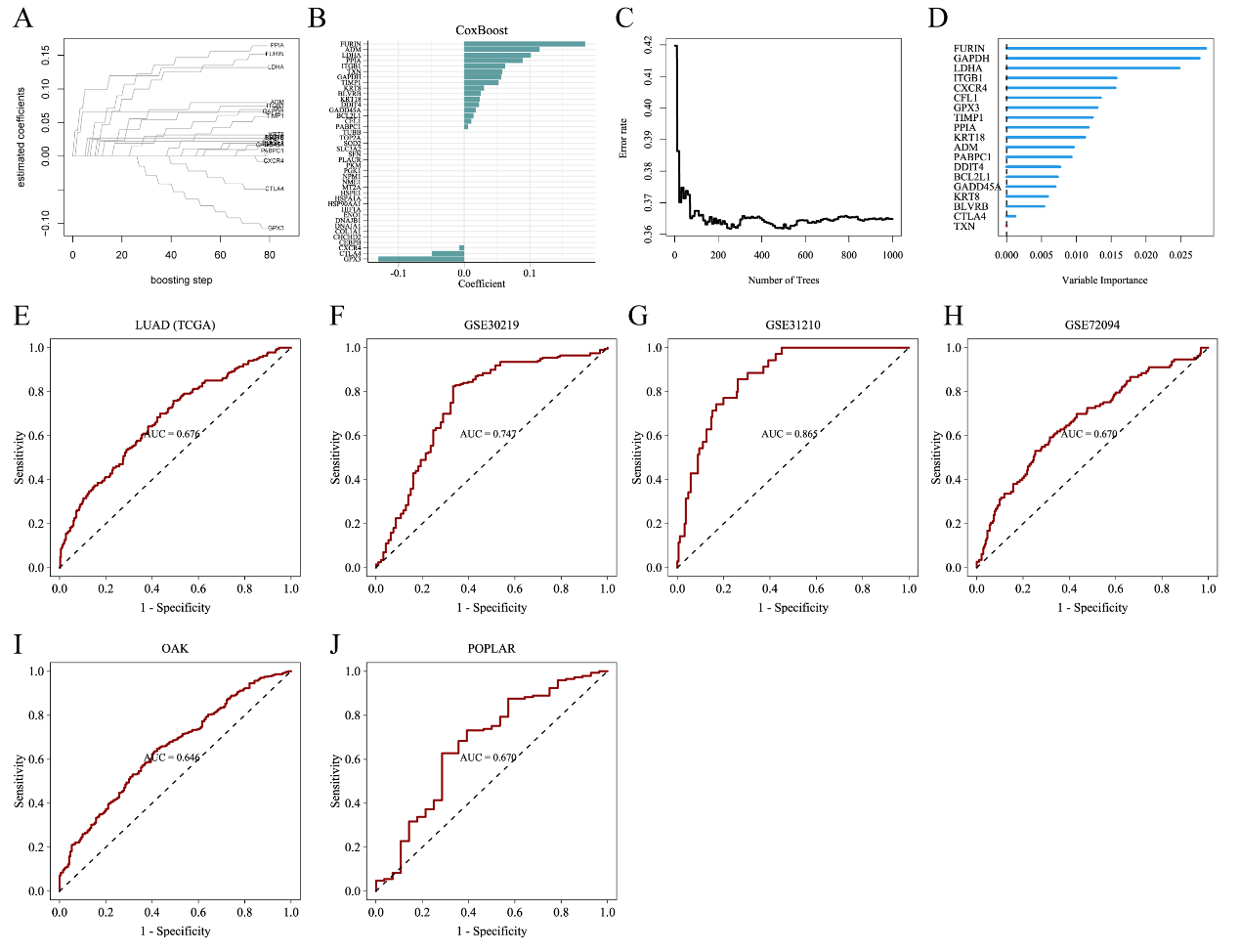


Supplementary Figure 1 | (A)-(B) CoxBoost for gene selection. (C)-(D) RSF for target gene selection. (E)-(J) Validation of the constructed scoring model’s prognostic performance using ROC curves and AUC in both training (TCGA-LUAD) and validation cohorts (GSE30219, GSE31210, GSE72094, OAK, POPLAR).


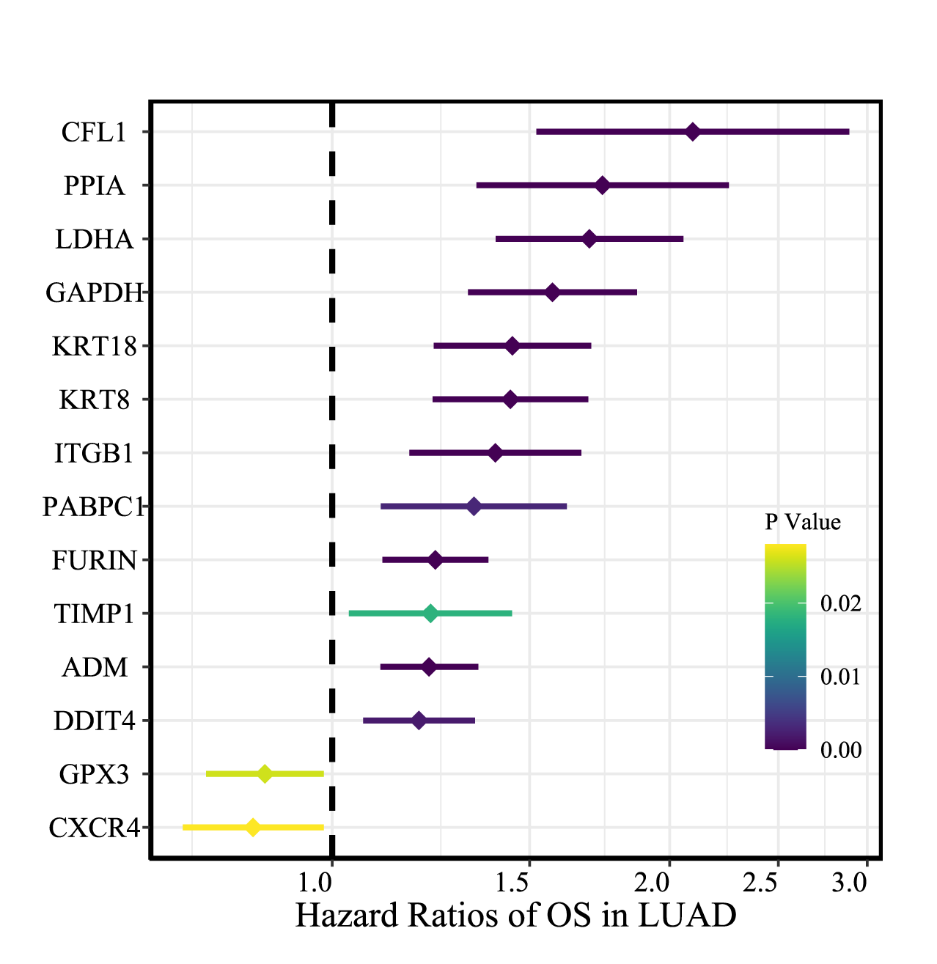


Supplementary Figure 2 | Prognostic correlation analysis of 14 selected modeling genes in the TCGA-LUAD cohort.


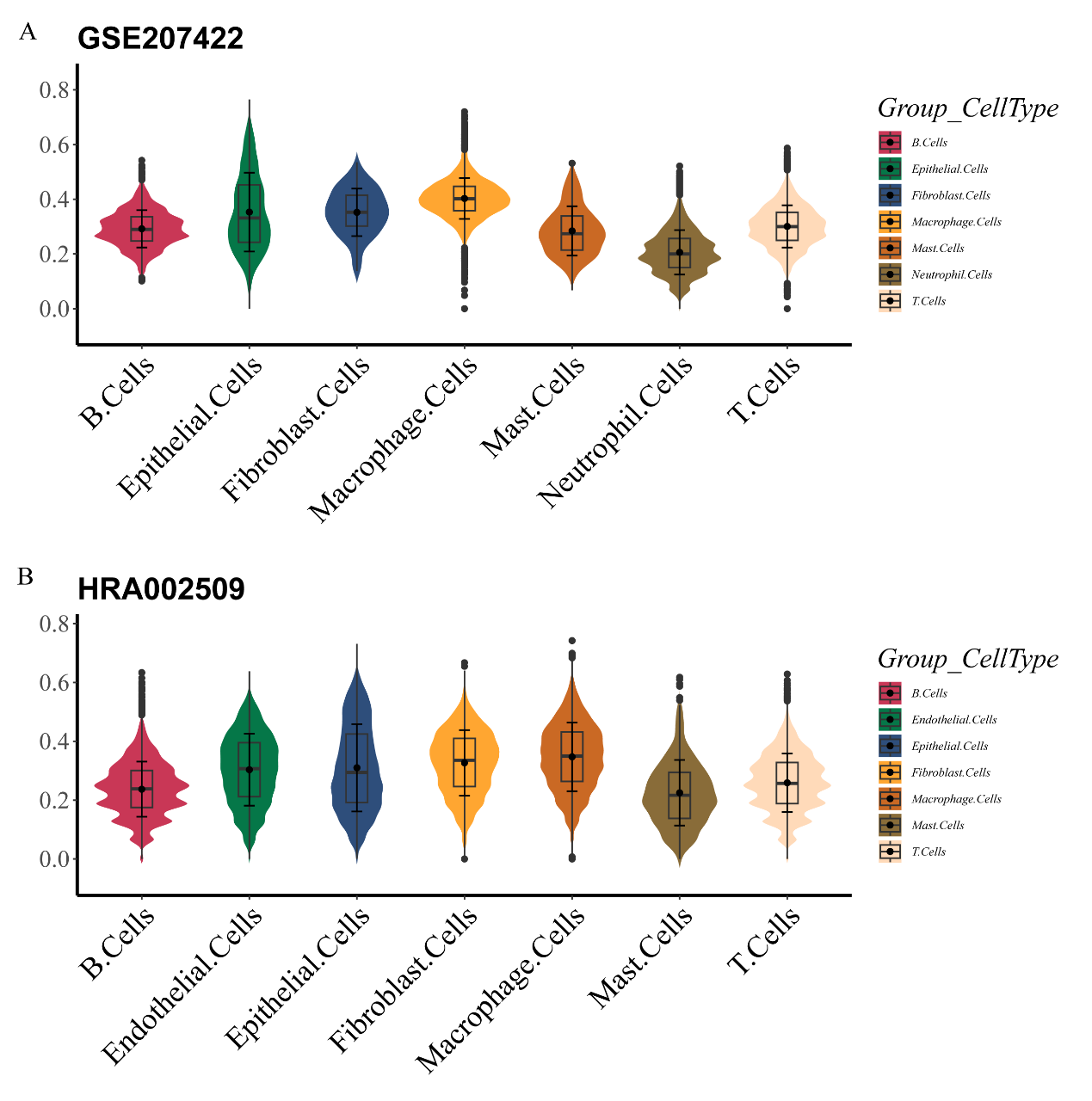


Supplementary Figure 3 | OSRGs scores for each cell subset in the GSE207422 and HRA002509 datasets.
